# A machine learning approach for genome-wide prediction of morbid and druggable human genes based on systems-level data

**DOI:** 10.1186/1471-2164-11-S5-S9

**Published:** 2010-12-22

**Authors:** Pedro R Costa, Marcio L Acencio, Ney Lemke

**Affiliations:** 1 Departamento de Física e Biofísica, Instituto de Biociências de Botucatu, UNESP - Univ Estadual Paulista, Distrito de Rubião Jr. s/n, Botucatu, São Paulo, 18618-970, Brazil

## Abstract

**Background:**

The genome-wide identification of both morbid genes, i.e., those genes whose mutations cause hereditary human diseases, and druggable genes, i.e., genes coding for proteins whose modulation by small molecules elicits phenotypic effects, requires experimental approaches that are time-consuming and laborious. Thus, a computational approach which could accurately predict such genes on a genome-wide scale would be invaluable for accelerating the pace of discovery of causal relationships between genes and diseases as well as the determination of druggability of gene products.

**Results:**

In this paper we propose a machine learning-based computational approach to predict morbid and druggable genes on a genome-wide scale. For this purpose, we constructed a decision tree-based meta-classifier and trained it on datasets containing, for each morbid and druggable gene, network topological features, tissue expression profile and subcellular localization data as learning attributes. This meta-classifier correctly recovered 65% of known morbid genes with a precision of 66% and correctly recovered 78% of known druggable genes with a precision of 75%. It was than used to assign morbidity and druggability scores to genes not known to be morbid and druggable and we showed a good match between these scores and literature data. Finally, we generated decision trees by training the J48 algorithm on the morbidity and druggability datasets to discover cellular rules for morbidity and druggability and, among the rules, we found that the number of regulating transcription factors and plasma membrane localization are the most important factors to morbidity and druggability, respectively.

**Conclusions:**

We were able to demonstrate that network topological features along with tissue expression profile and subcellular localization can reliably predict human morbid and druggable genes on a genome-wide scale. Moreover, by constructing decision trees based on these data, we could discover cellular rules governing morbidity and druggability.

## Background

Currently, the large-scale experimental identification of both morbid genes, i.e. those genes whose mutations cause hereditary human diseases, and druggable genes, i.e. genes coding for proteins whose modulation by small molecules elicits phenotypic effects, demands time-consuming and laborious approaches that are impractical for rapidly revealing the causal relationships between genes and diseases and determining the druggability of gene products. The discovery of morbid genes, for instance, requires a large effort to gather inheritance patterns from families with the disease and to perform linkage and mutation analyses in order to identify candidate gene(s) involved in a particular hereditary disorder [1]. In similar fashion, the discovery of new drug targets also requires a large effort involving a variety of genomics, proteomics, genetic association and forward and reverse genetics-related techniques [2] in order to find drugs capable to modulate disease processes.

In the light of above mentioned facts, a computational approach which could accurately predict morbid and druggable genes, especially on a genome-wide scale, would be thus invaluable since the number of experimental techniques to be performed to discover these genes could be minimized. With the vast amount of current available systems-level data, such as molecular interaction data and genome-wide gene expression and subcellular localization data, we have now the opportunity for developing a computational approach based on data mining tools, such as machine learning, to extract patterns that could be used as genome-wide predictors of morbid and druggable genes. Based on this assumption, we have previously used a machine learning-based methodology as a data mining tool to extract knowledge from systems-level data and then apply this knowledge to predict essential genes on a genome-wide scale and determine cellular rules for essentiality on *Escherichia coli*[3] and *Saccharomyces cerevisiae*[4]. In addition to attain successful prediction rates, we have also obtained biologically plausible cellular rules for gene essentiality using this machine learning approach.

Due this successful prediction of essential genes and determination of cellular rules for gene essentiality in *Escherichia coli* and *Saccharomyces cerevisiae*, we sought to verify in this present work whether a similar machine learning-based approach is able to predict human morbid and druggable genes on a genome-wide scale and to reveal cellular rules governing morbidity and druggability of genes. Using knowledge acquired from network topological features, tissue expression profile and subcellular localization data, we show here that the classifiers trained on these systems-level data can reliably predict morbid and druggable genes on a genome-wide scale and also can define some general rules governing morbidity and druggability in human.

## Results and discussion

### The integrated network of human gene interactions and calculation of topological features

For obtaining the network topological features used as training data for predicting morbid and druggable genes, we first constructed an integrated network of human gene interactions (INHGI) simultaneously containing experimentally verified protein physical interactions, metabolic interactions and transcriptional regulation interactions (definitions for each type of interaction are detailed in “Methods”). This network is comprised by 10,241 genes interacting with one another via 43,342 protein physical interactions, 24,540 metabolic interactions and 3,015 transcriptional regulation interactions. INHGI contains approximately 25% of the already identified ≈ 45,000 human genes according to the EntrezGene database [5].

From the INHGI, we calculated 12 different topological features for each gene, including degree centralities for each type of interaction, clustering coefficient, betweenness centralities for each type of interaction, closeness centrality and identicalness. The detailed description of these topological features and how they were calculated are found in the Additional file 1 and “Methods”.

### Evaluation of classifier performance

To examine how well a machine learning-based approach is able to predict human morbid and druggable genes on a genome-wide scale using knowledge acquired from systems-level data, we designed a meta-classifier similar to that used to predict essential genes in *Escherichia coli*[3] and *Saccharomyces cerevisiae*[4] and trained it on network topological features, tissue expression profile and subcellular localization data of known morbid and druggable genes (see “Methods” for details). We then assessed its performance by measuring its median recall, precision and area under the curve (AUC) of the receiver operating characteristic (ROC) curve across 10 different normal morbidity datasets and 10 different normal druggability datasets (see “Methods” for more details).

Before analyzing the performance measures of our meta-classifier trained on the datasets described above, we decided to estimate the performance measures of our meta-classifier on equivalent normal morbidity and druggability datasets where the class labels—morbid and druggable—were randomly shuffled among genes (shuffled morbidity and shuffled druggability datasets) and then compared them with our meta-classifier trained on the normal morbidity and druggability datasets. This was done to check whether the meta-classifier trained on non-shuffled datasets learned the traits actually associated with morbidity and druggability instead of traits associated with any random subset of genes. For this comparison, we used the Wilcoxon signed-rank statistical test as described in “Methods”. As can be observed in Table 1, all performance measures of our meta-classifier trained on the correspondent shuffled datasets were statistically different from measures of meta-classifier trained on normal datasets (for all performance measures, *W* ≤ *W_c_* with *N* = 10 at the p = 0.05 level; see “Methods” and [6]), thereby indicating that the traits actually associated with morbidity and druggability were learned by our meta-classifier.

**Table 1:** Classifier performance measures for prediction of morbid and druggable genes

Prediction of morbid genes													
Performance measure			Median [min,max] ^1^			Median [min,max] ^1^			*N*			*W*	*W_c_* (two-tailed *p* = 0.05)^2^

			Normal			Shuffled							
						
Precision			0.658 [0.648,0.679]			0.495 [0.473,0.522]			10			0	8 *
Recall			0.648 [0.632,0.657]			0.502 [0.471,0.521]			10			0	8 *
AUC			0.716 [0.706,0.729]			0.498 [0.462,0.526]			10			0	8 *

Prediction of druggable genes													

Performance measure			Median [min,max] ^1^			Median [min,max] ^1^			*N*			*W*	*W_c_* (two-tailed *p* = 0.05)^2^

			Normal			Shuffled						
						
Precision			0.748 [0.72,0.763]			0.5 [0.451,0.556]			10			0	8 *
Recall			0.782 [0.732,0.809]			0.492 [0.447,0.564]			10			0	8 *
AUC			0.820 [0.801,0.835]			0.500 [0.43,0.546]			10			0	8 *

^1^ Of 10 datasets

^2^ According to table of critical values for *W* in [6]

* Difference statistically significant

After confirmation that our meta-classifier trained on normal datasets was likely to learn the traits actually associated with morbidity and druggability, we aimed to analyze its performance measures. As shown in Table 1, for the genome-wide prediction of morbid genes, our meta-classifier achieved a median recall of 0.648 and a median precision of 0.658, i.e., it correctly recovered 64.8% of known morbid genes with a precision of 65.8%. Furthermore, the probability of a gene predicted as morbid belongs to the set of known morbid genes is 71.2% as indicated by the median AUC. For the genome-wide prediction of druggable genes, our meta-classifier achieved a median recall of 0.782 and a median precision of 0.748, i.e, it correctly recovered 78.2% of known druggable genes with a precision of 74.8% (Table 1). Furthermore, the probability of a gene predicted as druggable belongs to the set of known druggable genes is 82.0% as indicated by the median AUC.

The moderate values for both median recall (0.648) and median precision (0.662) for genome-wide prediction of morbid genes indicate that the level of noise in the training data is high and likely associated with existence of shared common features between morbid and non-morbid genes that induced our meta-classifier to yield a moderate performance in discriminating morbid from non-morbid genes. This could be partially due to the approach used to select non-morbid genes: since it is impossible at present to compile a list of genes not known to cause any hereditary disease, we selected genes not known to be morbid, i.e., all genes in INHGI except the known morbid genes, as non-morbid genes. Thus, some of these non-morbid genes may actually be existing unknown morbid genes sharing common characteristics with the existing known morbid genes. Other contributing factor for the existence of shared common features between morbid and non-morbid genes could be the incompleteness of INHGI: Stumpf *et al.*[7], for example, have estimated that the size of human interactome (only protein-protein interactions) is about 650,000 interactions. Since our network contains about 43,000 protein-protein interactions, we could envisage that the values of all network topological parameters might change with the enlargement of network size and, therefore, some of the network topological parameters-related shared common features between morbid and non-morbid might disappear as a consequence. The existence of shared common features between druggable and non-druggable genes also seems to affect the performance of our meta-classifier, but to a lesser extent: our meta-classifier achieved reliables values for the median recall (0.782) and precision (0.748) for genome-wide prediction of druggable genes (Table 1).

**Figure 1:**
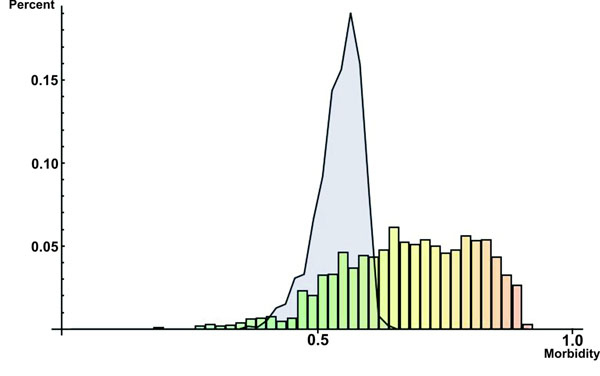
**Frequency distribution of known morbid genes per intervals of morbidity scores** Bars show the frequency distribution of known morbid genes (in percent) per 0.2 intervals of normal morbidity scores.The blue-shaded area represents the frequency distribution of known morbid genes (in percent) per intervals of shuffled morbidity scores.

**Figure 2:**
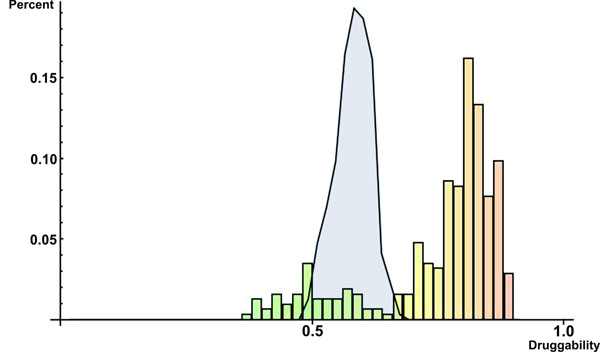
**Frequency distribution of known druggable genes per intervals of druggability scores** Bars show the frequency distribution of known druggable genes (in percent) per 0.2 intervals of normal druggability scores.The blue-shaded area represents the frequency distribution of known druggable genes (in percent) per intervals of shuffled druggability scores

Despite these limitations discussed above, our meta-classifier trained on network topological features, tissue expression profile and subcellular localization data seems indeed to be a reliable predictor of morbid and druggable genes on a genome-wide scale as shown by Figures 1 and 2: the frequency distribution of known morbid and known druggable genes per intervals of morbidity and druggability scores—probabilities of classifying genes as morbid and druggable, respectively, as output by the meta-classifier (see “Prediction of novel morbid and druggable genes” and “Methods” for more details)—tend to increase as morbidity (Figure 1) and druggability (Figure 2) scores increase.

### Evaluation of individual features on classifier performance

We sought to verify the influence of individual features on the meta-classifier performance. To achieve this goal, we first trained our meta-classifier on normal morbidity and druggability datasets without one of the features, which we call “without-one-feature” datasets as described in “Methods” . We then compared the output AUC values with those of meta-classifier trained on datasets with all features by using the Wilcoxon signed-rank statistical test [6]. A difference is considered statistically significant If the obtained *W* is lower than or equal to *W_c_* with a given *N* at the p = 0.05 level (see “Methods”). Note that we use AUC instead of recall or precision to compare the overall performances of meta-classifiers because it represents the meta-classifier performance across all combinations of recall and precision (see “Methods”). Table 2 shows that the median AUC of our meta-classifier trained on morbidity datasets without the number of tissues in which the gene is expressed at least 5 transcripts per million (tpm) (see “Methods” for details) was statistically lower than the median AUC for normal morbidity datasets (*W* = 7 *versus W_c_* = 8 for *N* = 10 at *p* = 0.05). So, the tissue expression profile seems to be an important feature to distinguish morbids from non-morbids genes.

**Table 2:** Statistical comparison of performances of classifiers trained on normal and without-one-feature morbidity datasets

Missing feature ^1^	Median AUC [min,max]^2^	N			*W*	*W_c_* (two-tailed *p* = 0.05)^3^
*ppi*	0.715 [0.705,0.726]	10			26	8
*metin*	0.714 [0.707,0.727]	10			26	8
*metout*	0.713 [0.707,0.729]	10			25	8
*regin*	0.714 [0.703,0.726]	9			18	6
*regout*	0.716 [0.705,0.729]	10			26	10
*c*	0.713 [0.701,0.724]	10			13	8
*identicalness*	0.711 [0.704,0.727]	10			24	8
*cent*	0.714 [0.707,0.727]	10			25	8
*inbet*	0.716 [0.708,0.731]	10			25	8
*inbetppi*	0.714 [0.707,0.727]	9			21	6
*inbetmet*	0.714 [0.707,0.728]	9			21	6
*inbetreg*	0.715 [0.706,0.727]	10			25	8
*numtissuesexp*^4^	0.709 [0.701,0.719]	10			7	8*
*avegexptec*^5^	0.715 [0.704,0.727]	10			27	8
Unknown	0.713 [0.701,0.725]	10			18	8
Cytoplasm	0.715 [0.706,0.728]	10			26	8
Endoplasmic reticulum	0.716 [0.705,0.727]	10			26	8
Mitochondrion	0.714 [0.706,0.728]	10			24	8
Nucleus	0.715 [0.704,0.728]	10			24	8
Other localization	0.714 [0.704,0.726]	10			21	8
Cellular component	0.714 [0.705,0.727]	9			21	6
Extracellular space	0.710 [0.7,0.723]	10			14	8
Golgi apparatus	0.715 [0.706,0.728]	10			26	8

Median AUC [min,max] for normal datasets: 0.716 [0.706,0.729]						

^1^ See “Methods” and Additional file 1 for a description of features

^2^ Of 10 datasets

^3^ According to table of critical values for *W* in [6]

^4^ The number of tissues (out of 32) in which the gene is expressed at least 5 transcripts per million (tpm) according to Reverter *et al*. [33]

^5^ The average expression in tpm among all the tissues in which the gene is expressed according to Reverter *et al*. [33]

* Difference statistically significant

As shown in Table 3, for prediction of druggable genes, the overall performance (AUC) of our meta-classifier was statistically lower following the removal of the plasma membrane feature (*W* = 1 *versus W_c_* = 8 for *N* = 10 at *p* = 0.05). This result is in concert with the most important cellular rule for druggability derived from the analysis of decision trees (see more details in “Methods”) that we will show in the section“Cellular rules for gene morbidity and druggability”): if proteins are located in plasma membrane, their encoding genes are likely to be druggable. This rule is supported by Bakheet and Doig [8] that demonstrated that proteins encoded by druggable genes had more transmembrane helices than proteins encoded by non-druggable ones which suggests that proteins encoded by druggable genes are more likely to be found in plasma membrane.

**Table 3:** Statistical comparison of performances of classifiers trained on normal and without-one-feature druggability datasets

Missing feature ^1^	Median AUC [min,max]^2^	*N*	*W*	*W_c_* (two-tailed *p* = 0.05)^3^
*ppi*	0.819 [0.798,0.835]	10	27	8
*metin*	0.817 [0.803,0.834]	10	26	8
*metout*	0.817 [0.801,0.832]	9	20	6
*regin*	0.818 [0.799,0.83]	9	18	6
*regout*	0.818 [0.801,0.833]	10	26	8
*c*	0.821 [0.799,0.836]	10	21	8
*identicalness*	0.819 [0.8,0.836]	10	27	8
*cent*	0.814 [0.797,0.832]	10	18	8
*inbet*	0.821 [0.804,0.837]	10	25	8
*inbetppi*	0.819 [0.803,0.833]	10	25	8
*inbetmet*	0.82 [0.791,0.833]	10	26	8
*inbetreg*	0.818 [0.802,0.83]	9	19	6
*numtissuesexp*^4^	0.806 [0.795,0.832]	9	11	6
*avegexptec*^5^	0.814 [0.799,0.835]	10	23	8
Unknown	0.816 [0.796,0.832]	9	12	6
Cytoplasm	0.814 [0.794,0.834]	10	20	8
Endoplasmic reticulum	0.820 [0.799,0.834]	10	27	8
Mitochondrion	0.820 [0.796,0.831]	9	22	6
Nucleus	0.816 [0.793,0.831]	10	20	8
Other localization	0.821 [0.802,0.837]	9	20	6
Cellular component	0.82 [0.801,0.835]	10	25	8
Extracellular space	0.817 [0.8,0.837]	10	26	8
Golgi apparatus	0.812 [0.8,0.834]	10	24	8
Plasma membrane	0.781 [0.762,0.816]	10	1	8*

Median AUC [min,max] for normal datasets : 0.820 [0.801,0.835]				

^1^ See “Methods” and Additional file 1 for a description of features

^2^ Of 10 datasets

^3^ According to table of critical values for *W* in [6]

^4^ The number of tissues (out of 32) in which the gene is expressed at least 5 transcripts per million (tpm) according to Reverter *et al*. [33]

^5^ The average expression in tpm among all the tissues in which the gene is expressed according to Reverter *et al*. [33]

* Difference statistically significant

### Comparison with other methods

Regarding prediction of morbid genes, there have been several methods available for predicting morbid genes [9-16]. However, our method can not be directly compared to most of them since they have been constructed to predict only small sets of disease-specific candidate genes, such as ENDEAVOUR [13] and ToppGene [15], while our method has been constructed for the genome-wide prediction of morbid genes. We can, however, compare our method to PROSPECTR [9], CIPHER [14] and that developed by Xu and Li [16]. Our method outperforms CIPHER (this method, for genome-wide prediction, yields a precision of about 0.1; there is no value of recall reported) and is comparable to PROSPECTR that achieves a recall of 0.70, a precision of 0.62 and an AUC of 0.70. Although PROSPECTR has a higher recall, we considered our method comparable to it as the precision and AUC values of our method are higher than those of PROSPECTR. Moreover, our performance measures are medians of 10 runs of 10-cross-fold validation (see “Methods” for more details), while the performance measures of PROSPECTR were obtained by only one run of 10-cross-fold validation.

The method developed by Xu and Li is the only genome-wide prediction method that apparently outperforms our method (this method achieves, for genome-wide prediction, an average recall about 0.78 and an average precision about 0.77). Their method is also based on network topological parameters, but while we trained our meta-classifier on various features, including 12 network topological parameters (see “Methods” and Additional file 1), they trained their classifiers on only five network topological parameters: degree, defined as the number of links to node *i*; 1N index, defined as the proportion of the number of links to morbid genes among all links to node *i*; 2N index, defined as the proportion of the number of links to morbid genes among all links to neighbors of node *i*; the average distance to morbid genes; and positive topological coefficient, a variant of the classical topological coefficient [17]. The apparent success of Xu and Li approach in predicting morbid genes mostly relies on the 2N index: when node *i* is a morbid gene, 2N index is always higher than zero since at least one neighbor of node *i*’s neighbor—the node *i* itself—is a morbid gene; if node *i* is a non-morbid gene, 2N index is higher than or equal to zero. Thus, this parameter induces a spurious correlation on dataset that is captured by classifiers that, in turn, achieve high performance measures. Therefore, the Xu and Li method can be disregarded for comparison purposes and, accordingly, our approach, although showing moderate recall and precision values, is currently, along with PROSPECTR, the most accurate predictor of morbid genes on a genome-wide scale.

Concerning the prediction of druggable genes, as for prediction of morbid genes, we can compare our method only with those developed to predict druggable genes on a genome-wide scale. Therefore, to our knowledge, we can compare our methodology with that developed by Sugaya and Ikeda [18]. Using support vector machines trained on 69 different features covering structural, drug and chemical, and functional information on protein-protein interactions, Sugaya and Ikeda classifiers achieved an average recall of 75%, an average precision of 70% and an average AUC of 72%, performance measures comparable to those obtained by our meta-classifier.

### Prediction of novel morbid and druggable genes

Since the morbidity and druggability of most of genes in INHGI are unknown—only ≈ 14% and ≈ 3% are known to be morbid and druggable, respectively—we applied our trained meta-classifier to determine the morbidity and druggability statuses of these genes. Instead of simply predicting genes as morbid or druggable, we decided to assign a “morbidity score” and a “druggability score” (see “Methods”) to each gene since we understand that there is no gene that is absolutely non-morbid or non-druggable. We also assigned to each gene a “shuffled morbidity score” and a “shuffled druggability score” to test the significance of normal scores. For this purpose, we used the Wilcoxon signed-rank statistical test as described in “Methods”.

Table 4 shows genes not known to be morbid with the 10 highest morbidity scores (see Additional file 2 for the normal and shuffled morbidity scores of all genes in INHGI). All these scores are significantly higher than the shuffled scores (*W* ≤ *W_c_* with *N* = 10 at the p = 0.05 level; see “Methods” and [6]). With the purpose of investigating whether the assigned scores resemble the potential morbidities of these genes, we mined the Human Genome Epidemiology Network (HuGENet) database [19] for articles clearly stating that such genes may be associated with some disease, which we call as “morbidity evidences”. According to this approach, we found that 10 of 11 (≈ 90%) genes with the 10 highest morbidity scores are considered to be associated with some disease (Table 4). This shows that our meta-classifier is quite capable of assigning high morbidity scores to genes potentially morbid.

**Table 4:** List of the human genes in the INHGI with the 10 highest morbidity scores

Gene			Morbidity score			(Median [min,max])^1^			*N*			*W*	*W_c_*^2^ (two-tailed *p* = 0.05)	Morbidity evidence^3^
			Normal			Shuffled								
							
TFRC			0.880 [0.576,0.939]			0.568 [0.447,0.678]			10			1	8*	5941956
ITGA5			0.875 [0.635,0.916]			0.491 [0.377,0.631]			10			0	8*	No evidence
LTF			0.868 [0.803,0.913]			0.509 [0.356,0.642]			10			0	8*	19258923
SFTPD			0.866 [0.618,0.923]			0.565 [0.458,0.682]			10			2	8*	19590686
THBS1			0.865 [0.831,0.918]			0.511 [0.354,0.566]			10			0	8*	18178577
TIMP2			0.860 [0.603,0.92]			0.574 [0.388,0.609]			10			0	8*	19933216
TGFB2			0.857 [0.565,0.918]			0.526 [0.407,0.707]			10			3	8*	19258923
CGA			0.856 [0.62,0.916]			0.535 [0.283,0.656]			10			0	8*	19730683
SPP1			0.856 [0.577,0.887]			0.564 [0.34,0.696]			10			0	8*	15868370
FLT1			0.854 [0.61,0.931]			0.527 [0.424,0.715]			10			3	8*	19741061
NOL3			0.850 [0.647,0.875]			0.576 [0.31,0.651]			10			1	8*	19773279

^1^ Of 10 scores

^2^ According to table of critical values for *W* in [6]

^3^ Pudmed IDs of most recent article(s) clearly stating a gene-disease association

* Difference statistically significant

Table 5 shows genes not known to be druggable with the 10 highest druggability scores (see Additional file 2 for the normal and shuffled druggability scores of all genes in INHGI). All these scores are significantly higher than the shuffled scores (*W* ≤ *W_c_* with *N* = 10 at the p = 0.05 level; see “Methods” and [6]). With the purpose of investigating whether the assigned scores resemble the potential druggabilities of these genes, we mined the literature for articles clearly stating that such genes may be drug target candidates, which we call as “druggability evidences”. According to this approach, we found that 8 of 11 (≈ 73%) genes with the 10 highest druggability scores are considered to be drug target candidates (Table 5). This shows that our meta-classifier is quite capable of assigning high druggability scores to genes potentially druggable. Among these candidates, five (*PLAU*, *CD8A,CD19*, *ITGAM* and *IL6*) are known morbid genes and two (*THBS1* and *TIMP2*) are within the list of genes with the 10 highest morbidity scores. About the known morbid genes with druggability evidence—*PLAU, CD19, ITGAM* and *IL6*—, it is interesting to note that the druggabilities assigned to these genes by our classifier are not related to the diseases caused by their corresponding mutated versions. The gene *PLAU* is a susceptibility gene for late-onset Alzheimer disease according to the Online Mendelian Inheritance in Man (OMIM) database [20] (MIM # 191840), but the protein encoded by this gene seems to be a good candidate target for treatment of cancer in combination with conventional therapeutics such as chemotherapy or radiation [21]. Similarly, mutations in the gene *CD19* cause antibody deficiency that increases susceptibility to infection ([22] (MIM #107265), but its encoded protein has proven to be a promise as a novel and well-tolerated therapy in B-cell non-Hodgkin’s lymphoma [23]. Regarding *ITGAM*, while Yang *et al.*[24] have confirmed the association of the this gene with disease susceptibility and renal nephritis of systemic lupus erythematosus (MIM # 609939), Romano *et al.*[25], on the other hand, have suggested that the protein encoded by *ITGAM* is a potential target of the femtomolar-acting eight-amino-acid peptide for protection against the deleterious effects of closed head injury in mice. Finally, according to OMIM database (MIM # 147620), the gene *IL6* mediates growth failure in Crohn disease [26], but we found that its encoded protein is a promising target for therapy of several chronic inflammatory and autoimmune diseases as well as in cancer [27]. These findings show that our classifier, besides discovering new druggable genes, can also reveal unexpectedly roles for known morbid genes in the modulation of diseases caused by other seemingly unrelated genes.

**Table 5:** List of the human genes in the INHGI with the 10 highest druggability scores

Gene			Druggability score			(Median [min,max])^1^			*N*			*W*	*W_c_*^2^ (two-tailed *p* = 0.05)	Druggability evidence^3^
			Normal			Shuffled								
							
HLA-F			0.887[0.803,0.915]			0.530[0.427,0.584]			10			0	8*	No evidence
PLAU^4^			0.886[0.808,0.907]			0.561[0.387,0.675]			10			0	8*	19301652
CD8A^4^			0.885[0.871,0.902]			0.56[0.37,0.664]			10			0	8*	No evidence
CD19^4^			0.880[0.751,0.907]			0.562[0.38,0.628]			10			0	8*	19509168
ITGAM^4^			0.878[0.614,0.887]			0.534[0.36,0.656]			10			1	8*	11931348
THBS1^5^			0.875[0.53,0.9]			0.532[0.293,0.592]			10			0	8*	17878288
ITGAX			0.873[0.784,0.897]			0.539[0.422,0.691]			10			0	8*	No evidence
CXCR5			0.871[0.755,0.895]			0.537[0.49,0.59]			10			0	8*	17652619
EBI3			0.871[0.801,0.888]			0.529[0.391,0.626]			10			0	8*	19556516
IL6^4^			0.87[0.766,0.893]			0.591[0.361,0.643]			10			0	8*	17465721
TIMP2^5^			0.869[0.645,0.916]			0.584[0.34,0.701]			10			0	8*	10985804

^1^ Of 10 scores

^2^ According to table of critical values for *W* in [6]

^3^ Pudmed IDs of most recent articles clearly stating that such genes may be drug target candidates

^4^ Morbid genes according to Morbid Map [46]

^5^ Genes among those with 10 highest morbidity scores (Table 4)

* Difference statistically significant

Two potential morbid genes, *THBS1* and *TIMP2*, reinforce the fact that our meta-classifier is able to reveal unexpectedly roles for morbid genes in the modulation of diseases caused by other seemingly unrelated genes. Mutations in the gene *THBS1* have been suggested to play a role in atherosclerosis and thrombosis [28], but its encoded protein may be considered a promising therapeutic target for diabetic nephropathy [29]; alterations in *TIMP2* has been demonstrated to be one of the causes of chronic obstructive pulmonary disease [30], but targeting its encoded protein may be a therapeutic intervention against connective amino acid tissue degradation [30].

### Cellular rules for gene morbidity and druggability

Beyond the prediction capability, machine learning techniques can be used for knowledge acquisition in order to describe patterns in datasets. The machine learning algorithms most used for knowledge acquisition are those that generate decision trees. Decision trees are decision support tools inferred from the training data that use a graph of conditions and their possible consequences. The structure of a decision tree consists of a root node representing the most important condition for discriminating classes, internal nodes representing additional conditions for class discrimination under the main condition, and leaf nodes representing the final classification. So, one can learn the conditions for classifying instances in a given class by following the path from the root node to the leaf node [31].

**Figure 3:**
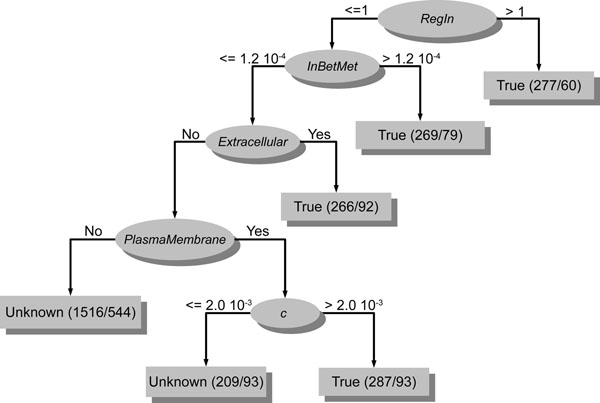
**Decision tree generated by training the J48 algorithm on the normal morbidity datasets** This decision tree was generated by training the J48 algorithm on the normal morbidity datasets (see “Methods”). The uppermost ellipse is the node root of tree that represents the most important condition for discriminating morbid genes from non-morbid genes. In this case, such condition is the number of transcription factors regulating the gene (*regin*). The remaining ellipses are internal nodes that represent additional conditions for considering a gene as morbid or non-morbid. In the left branch of tree, such conditions are a central position in a metabolic pathway (*inbetmet*), the extracellular or plasma membrane localization of respective encoded proteins and tendency of encoded proteins to form clusters with others (*c*). The rectangles depict genes that, under certain conditions (represented by the root node and internal nodes), are respectively and predominantly classified as morbid (**True**) and non-morbid (**Unknown**). In the round brackets inside rectangles, the number before the slash indicates the total number of genes that are actually morbid or non-morbid and the number after the slash indicates how many genes were incorrectly predicted.

**Figure 4:**
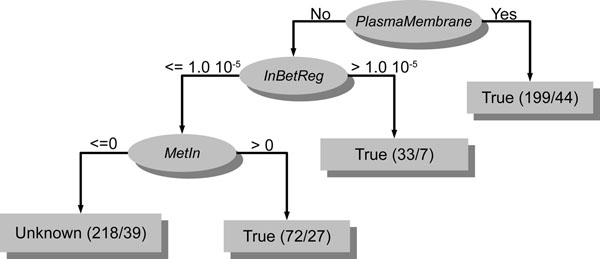
**Decision tree generated by training the J48 algorithm on the normal druggability datasets** This decision tree was generated by training the J48 algorithm on the normal druggability datasets (see “Methods”). The uppermost ellipse is the node root of tree that represents the most important condition for discriminating druggable genes from non-druggable genes. In this case, such condition is the plasma membrane localization of encoded proteins. The remaining ellipses are internal nodes that represent additional conditions for considering a gene as druggable or non-druggable. In the left branch of tree, such conditions are a central position in a transcriptional regulatory circuitry (*inbetreg*) and being an enzyme (*metin*). The rectangles depict genes that, under certain conditions (represented by the root node and internal nodes), are respectively and predominantly classified as druggable (**True**) and non-druggable (**Unknown**). In the round brackets inside rectangles, the number before the slash indicates the total number of genes that are actually druggable or non-druggable and the number after the slash indicates how many genes were incorrectly predicted.

Therefore, in order to discover the rules for gene morbidity and druggability, we analyzed decision trees generated by training the J48 algorithm, a WEKA’s implementation of the C4.5 algorithm [32] (for more details, see “Methods”), on the normal morbidity and druggability datasets containing all network topological features, tissue expression profiles and subcellular localization as training data. The decision trees in Figures 3 and 4 are the best representative tree among the 10 generated decision trees for morbidity (Figure 3) and the 10 generated decision trees for druggability (Figure 4).

From the best representative decision tree for morbidity, we were able to devise some general rules for morbidity in human. As we can observe in Figure 3, the root node of decision tree is the number of transcription factors that regulate a given gene (*regin*). So, this attribute can be considered the most important feature, among those used to train the J48 algorithm, for discriminating a morbid from a non-morbid gene. To reinforce this, we found, by walking the path from root node to first leaf node through the right branch, the following rule for morbidity: if genes are regulated by more than one transcription factor, they are likely to be morbid (Figure 3). The study by Reverter *et al.*[33] supports this rule as they showed that morbid genes are more likely to show tissue specific expression than non-morbid ones. Genes whose expression is tissue specific tend to be regulated by more transcription factors than those that are ubiquitously expressed, e.g. housekeeping genes, since a high level of transcriptional regulation is needed in this case.

Walking the path from root node to first and second leaf nodes through the left branch (Figure 4), we found the following rule for morbidity: if genes are regulated by one transcription factor and their encoded proteins are either centrally located in metabolic pathways (*inbetmet* is the betweenness centrality via metabolic interactions; see “Methods” and Additional file 1) or play a role in the extracellular region, genes are likely to be morbid. This rule is supported by Jimenez-Sanchez and colleagues [34] that showed that morbid genes are more likely to be enzymes than non-morbid ones and by Winter *et al*. [35] that demonstrated that ≈ 40% of proteins encoded by morbid genes are predicted to be secreted. Furthermore, if proteins are neither centrally located in metabolic pathways nor play a role in the extracellular region but are located in plasma membrane and tend to form clusters with other proteins (recall that *c* is the clustering coefficient, a network feature the measures the local group cohesiveness; see “Methods” and Additional file 1), their encoding genes are likely to be morbid. For this rule, we could not find any article supporting it. Therefore, the plasma membrane localization of proteins encoded by morbid genes as well as the tendency of these proteins to form clusters with other proteins are issues to be examined.

From the best representative decision tree for druggability, we were able to devise some general rules for druggability in human. As we can observe in Figure 4, the root node of decision tree is the plasma membrane localization of proteins. So, this attribute can be considered the most important feature, among those used to train the J48 algorithm, for discriminating a druggable from a non-druggable gene. To reinforce this, we found, by walking the path from root node to first leaf node through the right branch, the following rule for druggability: if proteins are located in plasma membrane, their encoding genes are likely to be druggable (Figure 4). This rule is supported by Bakheet and Doig [8] that demonstrated that proteins encoded by druggable genes had more transmembrane helices than proteins encoded by non-druggable ones which suggests that proteins encoded by druggable genes are more likely to be found in plasma membrane. Walking the path from root node to first and second leaf nodes through the left branch (Figure 4), we found the following rule for druggability: if proteins are not located in plasma membrane but are either centrally located in a transcriptional regulatory circuitry (*inbetreg* is the betweenness centrality via transcriptional regulation interactions; see “Methods” and Additional file 1) or are enzymes (*metin* is the number of metabolites catalyzed by a given enzyme; see Additional file 1), their encoding genes are likely to be druggable. This rule is partially supported by Bakheet and Doig [8] as they showed that druggable proteins are more likely to be enzymes than non-morbid ones. In respect to central position in a transcriptional regulatory circuitry, this is an issue that remains to be elucidated.

## Conclusions

The identification of morbid and druggable genes has largely been an experimental effort mostly performed by time-consuming experiments. In an effort to accelerate the pace of discovery of such genes, we designed a machine learning-based computational approach that relies on network topological features, tissue expression profile and subcellular localization information for predicting morbid and druggable genes in human on a genome-wide scale.

We could demonstrate that our method is able to reliable predict morbid and druggable genes on a genome-wide scale as demonstrated by (i) the moderate to high performance measures achieved by the meta-classifiers (Table 1), (ii) the observation that the designed meta-classifiers learned traits actually related to morbidity and druggability instead of traits associated with any random sets of genes (Table 1) and (iii) the fact that known morbid and druggable genes tend to have high morbidity and druggability scores, respectively (Figures 1 and 2). Furthermore, in comparison with other available genome-wide prediction methods, the performance of our method proved to be equal or superior . We could also devise some cellular rules for gene morbidity and druggability using all network topological features, tissue expression profile and subcellular localization information as learning attributes for generation of decision trees (see details in section “Cellular rules for gene morbidity and druggability”). We discovered that number of regulating transcription factors, the central position in metabolic pathways, the localization of their encoded proteins in extracellular region and plasma membrane and tendency to form clusters with other proteins are important factors determining gene morbidity. In respect to druggability, the important factors determining druggability are plasma membrane localization, a central position in a transcriptional regulatory circuitry and being an enzyme. The fact that almost all discovered rules are supported by some additional evidences solidifies decision trees as useful tools for extracting knowledge from complex biological data. Albeit the good prediction performance and the ability to discover cellular rules for morbidity and druggability, our approach suffers from three limitations. First, it depends on existing Gene Ontology annotation and interaction data which are likely to be enriched in small-scale experiments involving morbid and druggable genes. Second, the construction of an integrated network of gene interactions requires a large amount of experimental interaction data that are currently available only to a limited number of human genes—our INHGI, for example, covers only *≈* 25% of already identified human genes. Third, the lack of negative examples to train the classifier forces us to consider all genes not known to be morbid or druggable as *de facto* non-morbid and non-druggable genes. We expect, however, that such limitations will be soon addressed as more systems-level data are generated.

## Methods

### Generation of the set of training features

#### Network topological features

In order to compute the network topological features used as training features for predicting morbid and druggable genes, we first constructed an integrated network of human gene interactions (INHGI) based on assumption that two genes, *g*_1_ and *g*_2_, coding respectively for proteins *p*_1_ and *p*_2_, are interacting genes if *(i) p*_1_ and *p*_2_ interact physically (protein physical interaction), *(ii)* the transcription factor *p*_1_ directly regulates the transcription of gene *g*_2_, i.e., *p*_1_ binds to the promoter region of *g*_2_ (transcriptional regulation interaction), or *(iii)* the enzymes *p*_1_ and *p*_2_ share metabolites, i.e., a product generated by a reaction catalyzed by enzyme *p*_1_ is used as reactant by a reaction catalyzed by enzyme *p*_2_ (metabolic interaction). Experimentally verified human protein physical interactions data were obtained from the following databases: the Biological General Repository for Interaction Datasets (BioGRID) database (release 2.0.47; [36]), the Database of Interacting Proteins (DIP; release Hsapi20081014; [37]), the Human Protein Reference Database (HPRD; release 7; [1]), IntAct (release 91; [38]), the Molecular Interactions Database (MINT; October 2008 release; [39]) and The Munich Information Center for Protein Sequences (MIPS) Mammalian Protein Interaction Database (MPPI; downloaded in December 2008; [40]. Experimentally verified human transcriptional regulation interactions were obtained from the Transcriptional Regulatory Element Database (TRED; [41]).

Experimentally verified human metabolic interactions were extracted from the human metabolic model Recon 1 [42] by a code implemented in Mathematica® 7.0 (Wolfram Research, Inc.). We excluded those metabolic interactions generated by the so-called “currency metabolites”, abundant molecular species present throughout the cell most of the time and, therefore, unlikely to impose any constraints on the dynamics of metabolic reactions. Due to this feature of currency metabolites, the functionality of the network would be better represented without them [43]. We considered currency metabolites the eight most connected metabolites (ADP, ATP, H+, H_2_O, NADP+, NADPH, orthophosphate and pyrophosphate) in the original metabolic model Recon 1.

The final INHGI is the result of integration of the protein physical, metabolic and transcriptional regulation interactions datasets through genes common to these datasets. Before performing the integration, we converted all human gene names to their GeneIDs—as provided by the Entrez Gene database [5]—to avoid the creation of false interactions due to gene name ambiguity.

For each gene *g* in INHGI, we computed 12 network topological features as listed in Additional file 1. Briefly, degree centrality is defined as the number of links to node (in our case, gene). We considered each type of interaction as a distinct measure of degree as described in Additional file 1. Clustering coefficient (*c*) of a node (in our case, a gene) quantifies how close the node and its neighbors are to being a clique, i.e., all nodes connected to all nodes. For the INHGI, *c* is defined as the proportion of links between the genes within the neighborhood of *g* divided by the number of links that could possibly exist between them. Betweenness centrality reflects the role played by a node (in our case, a gene) in the global network architecture and, for the INHGI, is defined as the fraction of shortest paths between *g_i_* and *g_j_* passing through *g.* We computed the betweenness centrality based on shortest paths via all types of interaction (*inbet*) as well as based on shortest paths via each type of interaction (*inbetppi, inbetmet* and *inbetreg*)*.* Closeness centrality (*cent*) measures how close a node (in our case, a gene) is to all others in the network and, for the INHGI, is defined as the mean shortest path between *g* and all other genes reachable from it. Identicalness is the number of genes with identical network topological characteristics.

All these network topological features, except for the betweenness centrality-related features, were calculated by a program written in a Mathematica^®^ 7.0 notebook. The betweenness centrality-related features were calculated by the Python package *NetworkX* 0.99 [44].

#### Subcellular localization of human genes

We determined the subcellular localization of proteins encoded by the genes in the INHGI by using the QuickGO tool, a Gene Ontology (GO) browser associated with the integrated database resource for protein families (InterProt) at the European Bioinformatics Institute [45]. We selected GO slim terms—subsets of GO terms consisting of a limited number of high-level GO terms that cover some or all of the content of GO—related to cellular components provided by QuickGO to annotate genes in the INHGI. Genes were annotated to the following slim terms:“cytoplasm”, “endoplasmic reticulum”, “mitochondrion”, “nucleus”, “extracellular space”, “Golgi apparatus”, “plasma membrane” and “cellular component”. Genes annotated to other slim terms were reannotated to one of these terms or to a new term named “other localization” and genes with no GO cellular component slim term annotation was annotated to the term “unknown”.

#### Tissue expression profile of human genes

We retrieved the tissue expression profiles of genes in the INHGI from the study performed by Reverter and colleagues [33]. In their study, Reverter and colleagues mined three large datasets comprising expression data obtained from massively parallel signature sequencing across 32 tissues in order to classify genes as housekeeping or tissue-specific genes and then relate this tissue specificity with gene interactions and disease states. According to Reverter and colleagues, tissue expression profile of a given gene is (*i*) the number of tissues (out of 32) in which the gene is expressed at least 5 transcripts per million (tpm) and (*ii*) the average expression in tpm among all the tissues in which the gene is expressed [33].

### Classifier design and evaluation

#### Construction of training datasets

For evaluating the performance of the chosen training features–network topological features, subcellular localization and tissue expression profile–in predicting morbid and druggable genes, we constructed four different groups of balanced training datasets, i.e., datasets containing the same number of positive (in our case, morbid or druggable genes) and negative (in our case, non-morbid or non-druggable genes) examples: (1) “normal morbidity datasets”, (2) “shuffled morbidity datasets”, (3) “normal druggability datasets” and (4) “shuffled druggability datasets”.

For the construction of the morbidity datasets, we first gathered a list of “morbid genes”—genes whose mutations cause hereditary diseases—from the morbid map table in the Online Mendelian Inheritance in Man (OMIM) [46] and then mapped them to the INHGI. The final list of morbid genes used as positive examples to train our classifier is comprised by 1,412 morbid genes present in the INHGI. Regarding the negative examples, we considered as “non-morbid genes” the remaining genes present in the INHGI; this was done since building a list of genes not known to be involved in hereditary diseases is impossible currently. We randomly selected 10 different sets of 1,412 of these non-morbid genes and combine them with the list of morbid genes to build 10 different training datasets which we call “normal morbidity datasets”. From these normal morbidity datasets, we generate 10 different “shuffled morbidity datasets” by randomly shuffling the class labels (morbid and non-morbid) among genes.

For the construction of the druggability dataset, we first built a list of “druggable genes”—genes coding for proteins whose modulation by small molecules elicits phenotypic effects—from the drug-target network constructed by Yildirim and colleagues [47] and then mapped them to the INHGI. The final list of druggable genes used as positive examples to train our classifier is comprised by 257 druggable genes present in the INHGI. Regarding the negative examples, we considered as “non-druggable genes” the remaining genes present in the INHGI; this was done since, similar to non-morbid genes, it is also impossible to construct a list of genes coding for proteins whose modulation by small molecules do not elicits phenotypic effects. We randomly selected 10 different sets of 257 of these non-druggable genes and combine them with the list of druggable genes to build 10 different training datasets which we call “normal druggability datasets”. From these normal druggability datasets, we generate 10 different “shuffled druggability datasets” by randomly shuffling the class labels (druggable and non-druggable) among genes. We also constructed 25 additional morbidity and 25 additional druggability datasets lacking one of the 25 features used as training attributes. We call these datasets as “without-one-feature” datasets, where *one* can be replaced by the name of feature.

#### Classifier design

Using WEKA (Waikato Environment for Knowledge Analysis) software package, a collection of machine learning algorithms for data mining tasks [48], we designed the classifier used for predicting morbid and druggable genes in the INHGI. This classifier is an ensemble of seven decision tree algorithms using the meta-classifier “Vote”, a WEKA’s implementation of the voting algorithm that combines the output predictions of each classifier by different rules [49]. We combined the classifiers by the average rule, where the output predictions computed by the individual classifiers for each class are averaged and this average is used in its decision [49]. The classifiers composing our model were: (1) REPtree [48], (2) random tree [48], (3) random forest [50], (4) J48, a WEKA’s implementation of the C4.5 decision tree [32], with minimum number of 32 instances per leaf, (5) best-first decision tree with minimum number of 32 instances at the terminal nodes [51], (6) logistic model tree [52] and (7) alternating decision tree with 25 boost iterations [53]. In addition, we applied the bootstrap aggregating (bagging) approach [54] to each classifier. Parameters values for each classifier are provided in the Additional file 3.

#### Classifier evaluation

We assessed the performance of our classifier by estimating the following measures: recall, precision and area under the curve (AUC) of the receiver operating characteristic (ROC) curve. Recall is the proportion of actual morbid or druggable genes which are correctly predicted as such against all actual morbid or druggable genes:

TP (true positive) denotes the amount of actual morbid or druggable genes correctly predicted as such and FN (false negative) denotes the amount of actual morbid or druggable genes incorrectly predicted as non-morbid or non-druggable, respectively.

Precision is the proportion of actual morbid or druggable genes which are correctly predicted as such against all genes predicted as morbid or druggable:

FP denotes the amount of actual non-morbid or non-druggable genes incorrectly predicted as morbid or druggable, respectively.

The AUC is a widely used summary measure of the ROC curve–a plot of the true positive rate versus false positive rate that indicates the probability of a true positive prediction as a function of the probability of a false positive prediction for all possible threshold values [55]–and is equivalent to the probability that a randomly chosen negative example (in our case, a non-morbid or non-druggable gene) will have a smaller estimated probability of belonging to the positive class than a randomly chosen positive example (in our case, a morbid or druggable gene) [56].

We estimated the above-mentioned performance measures by performing a 10-fold cross-validation test–using WEKA–on the 10 normal and 10 shuffled morbidity datasets and on the 10 normal and 10 shuffled druggability datasets constructed as described in the section “Construction of training datasets”. During the 10-fold cross-validation test process, each dataset is randomly partitioned into 10 subsets. Of the 10 subsets, a single subset is retained as the validation data for testing the model, and the remaining 9 subsets are used as training data. The cross-validation process is then repeated 10 times, with each of the 10 subsets used exactly once as the validation data. The 10 results from the folds are then averaged to produce a single estimation for each performance measure for each dataset. We reported the performance measures estimated by the 10-fold cross-validation as medians of the 10 datasets for each category (normal morbidity, shuffled morbidity, normal morbidity and shuffled morbidity).

The statistical comparisons of (i) the performance measures estimated by our classifier trained on normal and shuffled datasets, (ii) the AUC values estimated by our classifier trained on normal datasets and without-one-feature datasets, and (iii) the normal and shuffled morbidity and druggability scores for each gene in INHGI were performed by the Wilcoxon signed-rank test [6]. Following established conventions in the machine learning community, we used this test since it makes minimal assumptions about the underlying distribution of performance measures used to evaluate classifiers [57]. The differences were statistically significant if the obtained Wilcoxon’s test statistic value (*W*) was equal to or smaller than the critical Wilcoxon’s test statistic value (*W_c_*) for a given sample size (*N*) at the two-tailed significance level of 0.05 (*p* = 0.05) according to the table of critical values for the Wilcoxon test [6].

### Prediction of novel morbid and druggable genes

The “normal morbidity scores” and the “normal druggability scores” were generated by applying the models constructed by training our meta-classifier on the normal datasets to the entire set of genes in INHGI where the class labels were removed. These scores are the probability values of classifying each gene as morbid or druggable as returned by the models. The final normal morbidity and druggability scores are median scores of 10 scores. We also obtained “shuffled morbidity scores” and “shuffled druggability scores” that were generated by models trained on the shuffled datasets.

### Determination of rules for gene morbidity and druggability

The determination of rules for gene morbidity and druggability was performed by analyzing the best representative decision tree for each category among the 10 decision trees generated through the training of J48 algorithm [32] on the 10 normal morbidity and 10 normal druggability datasets. The parameters values for producing decision trees by J48 algorithm training are provided in the Additional file 3.

## Competing interests

The authors declare that they have no competing interests.

## Authors contributions

PRC obtained the tissue expression profile and gene ontology data, analyzed the meta-classifiers’ performances, implemented the program for calculation of network topological features and drafted the manuscript. MLA obtained all interaction data, constructed the network, designed the meta-classifier, pursued the biological interpretation of results and drafted the manuscript. NL conceived, designed and directed the project. All authors read and approved the final manuscript.

## Supplementary Material

Additional file 1 **Network topological features** Description: This file includes a table showing the functions and descriptions of the 12 network topological features used as learning attributes for training the classifier algorithm Click here for file

Additional file 2 **Morbidity and druggability scores of genes in INHGI** Description: Tab-limited text file containing all genes (Entrez GeneIDs) in the INHGI with their morbidity and druggability scores. Click here for file

Additional file 3 **Parameters used to train the meta-classifier and J48** Description: File containing all parameters values used to train the meta-classifier for prediction of morbid and druggable genes and all parameters values used to train the J48 algorithm to generate decision trees for discovery of cellular rules for morbidity and druggability. Click here for file
