# Supplementary material for: A machine learning approach for genome-wide prediction of morbid and druggable human genes based on systems-level data
Source: BMC Genomics. 2010 Dec 22;11(Suppl 5):S9. doi: 10.1186/1471-2164-11-S5-S9 (PMC3045802; doi:10.1186/1471-2164-11-S5-S9)
Supplement: Additional file 3 — Parameters used to train the meta-classifier and J48 Description: File containing all parameters values used to train the meta-classifier for prediction of morbid and druggable genes and all parameters values used to train the J48 algorithm to generate decision trees for discovery of cellular rules for morbidity and druggability. [file 1471-2164-11-S5-S9-S3.pdf]

A. Parameters (**) of the decision tree-based meta-classifier used to predict morbid and druggable genes (based on the graphic user interface of WEKA software package):

Meta-classifier: Vote
**classifiers: 8 weka.classifiers.Classifier (decision tree classifiers with bootstrap aggregating [bagging] approach as described below)
**combinationRule: Average of Probabilities
**debug: False
**seed: 1

-- We applied the class "weka.classifiers.meta.Bagging" (bootstrap aggregating [bagging]) approach to each classifier (see details in the section "Methods" in paper) with the following parameters:
**bagSizePercent: 100
**calcOutOfBag: false
**classifier: one of the 7 decision tree classifiers whose parameters are described below.
**debug: False
**numiterations: 20
**seed: 1

Parameters of the 7 bagged decision tree classifiers:

1. REPTree
**debug: False
** maxDepth: -1
** minNum: 2.0
**minVarianceProp: 0.0010
**noPrunning: False
**numFolds: 3
**seed: 1

2. Random Tree
**KValue: 1
**debug: false
**maxDepth: 0
**minNum: 1.0
**seed: 1

3. Random Forest
**debug: False
**maxDepth: 0
**numFeatures: 0
**numTrees: 10
seed: 1

4. J48
**binarySplits: False
** confidenceFactor: 0.5
**debug: False
**minNumObj: 32
**numFolds: 3
**reducedErrorPruning: False
**saveInstanceData: False
**seed: 1
**unpruned : False
**useLaplace: False

5. Best-first decision tree (BFtree)
**debug: False
**heuristic: True
**minNumObj: 32

**numFoldsPruning: 5
**pruningStrategy: Post-pruning
**seed: 1
**sizePer: 1.0
**useErrorRate: True
**useGini: True
**useOneSE: False

6. Logistic model tree (LMT)
**convertNominal: False
**debug: False
**errorOnProbabilities: False
**fastRegression: True
**minNumInstances: 15
**numBoostingIterations: -1
**splitOnResiduals: False
**useAIC: False
**weightTrimBeta: 0.0

7. Alternating decision tree (ADtree)
**debug: False
**numOfBoostingIterations: 25
**randomSeed: 0
**saveInstanceData: False
**searchPath: Expand the heaviest path

B. Parameters (**) of the J48 classifier used to generated the decision trees for determination of cellular rules for morbidity and druggability (based on the graphic user interface of WEKA software package):

**binarySplits: False
** confidenceFactor: 0.5
**debug: False
**minNumObj: 128 (morbidity) or 32 (druggability)
**numFolds: 3
**reducedErrorPruning: False
**saveInstanceData: False
**seed: 1
**unpruned : False
**useLaplace: False
